# Photodynamic Therapy for Atherosclerosis: Past, Present, and Future

**DOI:** 10.3390/pharmaceutics16060729

**Published:** 2024-05-29

**Authors:** Yanqing Lin, Ruosen Xie, Tao Yu

**Affiliations:** 1 Ultrasound in Cardiac Electrophysiology and Biomechanics Key Laboratory of Sichuan Province, Sichuan Provincial People’s Hospital, University of Electronic Science and Technology of China, Chengdu 610072, China; linyanqing@med.uestc.edu.cn; 2 Wisconsin Institute for Discovery, University of Wisconsin–Madison, Madison, WI 53705, USA; rxie23@wisc.edu; 3 Department of Cardiac Surgery, Sichuan Provincial People’s Hospital, University of Electronic Science and Technology of China, Chengdu 610072, China

**Keywords:** photodynamic therapy, atherosclerosis, plaque, nanoparticles

## Abstract

This review paper examines the evolution of photodynamic therapy (PDT) as a novel, minimally invasive strategy for treating atherosclerosis, a leading global health concern. Atherosclerosis is characterized by the accumulation of lipids and inflammation within arterial walls, leading to significant morbidity and mortality through cardiovascular diseases such as myocardial infarction and stroke. Traditional therapeutic approaches have primarily focused on modulating risk factors such as hypertension and hyperlipidemia, with emerging evidence highlighting the pivotal role of inflammation. PDT, leveraging a photosensitizer, specific-wavelength light, and oxygen, offers targeted treatment by inducing cell death in diseased tissues while sparing healthy ones. This specificity, combined with advancements in nanoparticle technology for improved delivery, positions PDT as a promising alternative to traditional interventions. The review explores the mechanistic basis of PDT, its efficacy in preclinical studies, and the potential for enhancing plaque stability and reducing macrophage density within plaques. It also addresses the need for further research to optimize treatment parameters, mitigate adverse effects, and validate long-term outcomes. By detailing past developments, current progress, and future directions, this paper aims to highlight PDT’s potential in revolutionizing atherosclerosis treatment, bridging the gap from experimental research to clinical application.

## 1. Introduction

Atherosclerosis is a major cause of death worldwide, surpassing infectious diseases as the foremost public health issue [1,2,3]. This disease is primarily caused by the buildup of low-density lipoprotein (LDL) and inflammation in the arteries, leading to serious health problems such as heart attacks, strokes, and peripheral artery disease. Recent research has highlighted the role of inflammation in the development and progression of atherosclerosis, suggesting that targeting inflammatory processes could be an effective strategy for treating this condition [4,5,6]. Traditional treatment for atherosclerosis typically involves a combination of lifestyle modifications and pharmacological interventions. Lifestyle changes include adopting a heart-healthy diet low in saturated and trans fats, engaging in regular physical activity, maintaining a healthy weight, and quitting smoking [7]. Pharmacological treatments may include statins to lower LDL cholesterol levels, antiplatelet drugs such as aspirin to prevent blood clots, and antihypertensive medications to control blood pressure [8,9,10]. In more severe cases, surgical procedures such as angioplasty and stenting or coronary artery bypass grafting may be necessary to improve blood flow to the heart [11]. The goal of traditional treatment is to slow the progression of atherosclerosis, reduce the risk of cardiovascular events, and manage the symptoms associated with the condition [12].

PDT is an emerging treatment that uses light-activated photosensitizers to produce reactive oxygen species, offering a targeted approach to treating atherosclerotic lesions [13,14]. This review aims to explore the importance of developing treatments for atherosclerosis, with a focus on PDT as a therapeutic option. In this review, we will discuss the mechanisms by which PDT works, its application in clinical settings, and its potential to improve the management of cardiovascular diseases. Our goal is to provide a clear and comprehensive overview of the current state of research in this area and to identify future directions for the development of effective treatments for atherosclerosis.

## 2. Atherosclerosis: An Overview

**Figure 1:**
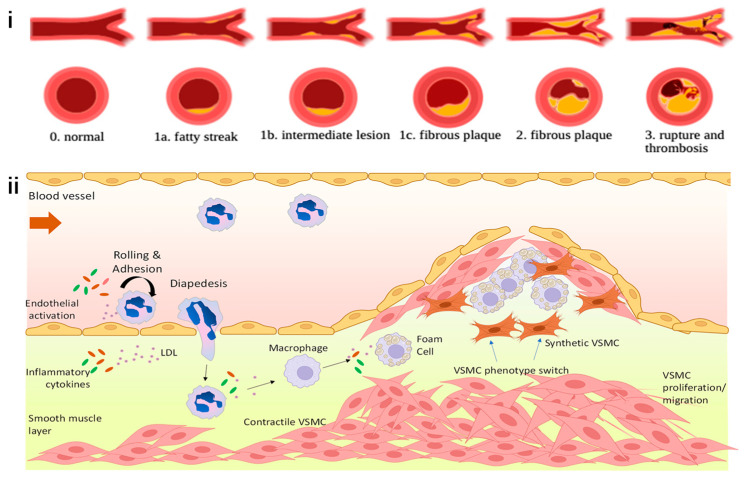
The mechanism of atherosclerosis development. (**i**) The individual stages of atherosclerosis are fatty streak, intermediate lesion, fibrous plaque, rupture, and thrombosis. Ref. [19], Copyright 2024, MDPI. (**ii**) Atherosclerotic lesions develop at the site of vascular endothelium damage, leading to up-regulation of vascular adhesion molecules (e.g., VCAM1, ICAM) and expression of monocyte chemoattractants [monocyte chemoattractant protein-1 (e.g., MCP-1/CCL2)]. Together, these facilitate leukocyte migration and adhesion. Smooth muscle cells (vSMCs) proliferate and migrate to the lesion, undergoing a defifferentiation from contractile to synthetic SMCs. Recent studies investigating SMC plasticity during atherosclerosis suggest that SMC phenotypic switching can contribute multiple cell types within the lesion, including macrophage-like cells, foam-cell-like cells, myofibroblast-like cells, and mesenchymal stem cell-like cells. In response to proatherogenic stimuli within the vessel wall, macrophages transform into lipid-laden foam cells, which significantly contribute to lesion progression. T-cell activation and platelet adherence also occur. Plaque rupture occurs in advanced lesions as a result of fibrous cap thinning. Ref. [35], Copyright 2022, Portland Press.

Atherosclerosis has evolved as a leading cause of death and disability globally, surpassing communicable diseases to become the top health concern [2,15,16]. Fueled by the accumulation of low-density lipoprotein (LDL) and other lipoproteins in arteries, it is primarily driven by inflammation, which orchestrates its progression and outcome [17,18,19] (Figure 1). Traditional non-invasive therapeutic interventions have targeted risk factors such as hypertension and hypercholesterolemia [20,21]. However, the discovery that inflammation mediates many risk factors has paved the way for targeting inflammatory pathways as a new avenue to prevent and treat the disease. This approach has been validated by clinical studies such as the Canakinumab Anti-inflammatory Thrombosis Outcomes Study (CANTOS), which demonstrated that modulation of inflammation can forestall the clinical complications of atherosclerosis [22]. Smooth muscle cells (SMCs), macrophages, and neutrophils are among the key cells involved in its process [23,24,25,26,27,28,29,30,31,32,33]. Distinct cellular architectures, such as resident arterial macrophages and differentiated yet plastic medial vascular smooth muscle cells, contribute to atherosclerotic plaque development [23,34,35]. Obesity, diabetes, hypertension, and hypercholesterolemia are significant risk factors that exacerbate the risk [36,37,38,39]. Emerging evidence has also identified nontraditional drivers such as disturbed sleep, the microbiome, environmental stress, and physical inactivity. Atherosclerotic cardiovascular disease manifests in various ways, including myocardial infarction, stroke, and peripheral arterial disease, contributing significantly to morbidity and mortality worldwide [40,41,42,43]. The inflammatory nature of this disease highlights the need for therapeutic interventions that target inflammatory pathways [44,45,46,47,48].

Traditional diagnostic techniques for atherosclerosis involve a combination of imaging studies and blood tests that aim to assess the arterial walls for signs of plaque accumulation and evaluate the risk factors associated with the disease [49]. Imaging studies such as ultrasound, computed tomography (CT) scans, and magnetic resonance imaging (MRI) provide direct visualization of arterial plaque and help in assessing the degree of stenosis or narrowing of the arteries [50,51,52,53]. These methods enable the evaluation of plaque characteristics, such as composition and morphology, which are crucial in determining the vulnerability of the plaque to rupture—a primary cause of acute cardiovascular events such as myocardial infarction and stroke. Additionally, angiography, particularly coronary angiography, remains a gold standard for visualizing the inner lumen of coronary arteries, despite being invasive [54]. This technique helps in identifying significant blockages that might require interventions such as angioplasty or stent placement. However, angiography primarily reveals the arterial lumen size rather than giving details about the arterial wall or the plaque itself. Blood tests play a complementary role by evaluating lipid profiles (including total cholesterol, LDL cholesterol, HDL cholesterol, and triglycerides) and markers of inflammation (such as C-reactive protein). Elevated levels of LDL cholesterol are directly implicated in the pathogenesis of atherosclerosis, while elevated markers of inflammation indicate an ongoing inflammatory process within the arterial walls, contributing to plaque development and progression [55]. Despite the utility of these diagnostic tools, there are limitations and challenges to early detection. Traditional imaging techniques might not adequately predict plaque rupture risk since they focus more on the degree of arterial stenosis rather than plaque vulnerability. Moreover, blood tests for lipid levels and inflammatory markers provide a generalized, systemic assessment and may not directly reflect the localized pathology within the arterial walls. Therefore, there is a growing interest in developing more sensitive and specific biomarkers and imaging modalities that can better predict the risk of plaque rupture and subsequent cardiovascular events [56]. Recent advancements aim to address these challenges by focusing on identifying features of vulnerable plaque, including lipid-rich necrotic core, thin fibrous cap, and active inflammation, using more sophisticated imaging techniques such as intravascular ultrasound (IVUS), optical coherence tomography (OCT), and positron emission tomography (PET) combined with CT (PET-CT) (Table 1). Furthermore, research into novel biomarkers that more specifically reflect the biological activity of atherosclerotic lesions is ongoing to improve the precision of risk assessment and the early identification of individuals at high risk for acute cardiovascular events.

The increasing prevalence of atherosclerosis globally has necessitated the development and refinement of treatments targeting the condition directly and its risk factors. The traditional focus has been on non-invasive pharmacological interventions aimed at modulating risk factors such as hypertension and hyperlipidemia. However, emerging evidence underscores the central role of inflammation, from atherosclerosis initiation to the manifestation of complications. Thus, targeting inflammatory pathways presents a novel and promising avenue for the prevention and treatment of atherosclerosis. The realization that inflammation plays a driving role in atherosclerosis—interconnecting traditional risk factors with altered artery wall cell behavior and leukocyte recruitment—has opened various therapeutic targets. Recent clinical studies, notably the CANTOS, and trials on low-dose colchicine, have demonstrated that modulating inflammation can indeed forestall the clinical complications of atherosclerosis. These findings anchor the need for continuous preclinical research aimed at refining anti-inflammatory strategies suitable for human disease intervention. Among notable therapeutic targets in the realm of innate immunity are the NLRP3 inflammasome and its associated cytokines IL-1β, IL-18, and IL-6, indicating promising pathways for intervention [57,58,59,60,61]. Anti-inflammatory therapies such as IL-1β neutralizing agents have shown the potential to improve outcomes in patients with good LDL control but still at risk for heart failure [62,63,64]. This highlights the crucial balance needed in targeting inflammation to optimize the benefit-risk ratio and prevent impairing host defenses. Furthermore, advances in nanomedicine offer opportunities to enhance the safety and effectiveness of therapies by enabling targeted drug delivery to specific sites within the vascular system [65,66,67]. This can potentially reduce systemic side effects associated with broad inhibition of inflammatory pathways. Lifestyle interventions, encompassing physical activity, diet modification, and adherence to a healthy lifestyle, have also shown significant potential for reducing cardiovascular risk. These modifications not only address traditional risk factors but also modulate inflammatory processes, indicating their essential role in a comprehensive strategy against atherosclerosis.

## 3. Photodynamic Therapy in Atherosclerosis

PDT represents a novel and minimally invasive treatment strategy for a variety of diseases, including cardiovascular conditions such as atherosclerosis and various oncological diseases [68,69,70,71]. Central to the PDT process are three essential components: A photosensitizer (PS), light of a specific wavelength to activate the PS, and oxygen within the target tissues. The procedure begins with the introduction of a photosensitizer into the body, where it preferentially accumulates within target cells or tissues, including subcellular organelles such as mitochondria, lysosomes, and plasma membranes [72,73,74,75,76]. This specificity minimizes damage to surrounding healthy tissue and underscores PDT’s targeted approach. Upon illumination with appropriate light, the photosensitizer enters an excited state, initiating the generation of reactive oxygen species (ROS) through type I and type II photophysical reactions. These ROS, capable of inducing cell death via apoptosis or necrosis, highlight the mechanism through which PDT exerts its therapeutic effects [77,78,79]. The exact pathways vary depending on factors such as the type of cell, the photosensitizer used, its cellular localization, and the light’s dosage and characteristics [80]. The mechanism involves direct killing of diseased cells, damage to their vasculature leading to nutrient deprivation, and stimulation of an immune response [81,82,83,84]. The specificity of PDT, especially for cardiovascular diseases such as atherosclerosis, hinges on the precise localization and accumulation of the photosensitizer within the target tissues [85]. Atherosclerosis, characterized by lipoprotein deposition, inflammatory cell infiltration, and plaque buildup in arterial walls, is a major cardiovascular morbidity and mortality contributor [86]. PDT operates through the selective accumulation of photosensitizers in atherosclerotic plaques, targeting diseased cells and sparing healthy tissues. This selective cytotoxicity offers a promising avenue distinct from traditional systemic risk factor modulation strategies. The efficacy of PDT in preclinical studies, including reductions in macrophage density within plaques and enhanced plaque stability, depends on the photosensitizer’s specificity, optimal light dosimetry, and effective target site reach within vascular structures. The exploration of nanoparticles to improve photosensitizer delivery and specificity further exemplifies the innovative strides in PDT for atherosclerosis, highlighting the potential for increased efficacy and precision with reduced systemic toxicity [87]. As PDT continues to evolve, it stands on the cusp of transitioning from an experimental approach to a viable adjunct for treating atherosclerosis. The integration of targeted therapy, minimally invasive delivery, and plaque stabilization positions PDT as a beacon of innovation, promising a future where cardiovascular health benefits from the precision and efficacy of light-based interventions. However, transitioning from preclinical promise to clinical application requires rigorous investigation, including optimizing treatment parameters, mitigating potential adverse effects, and validating long-term efficacy and safety through comprehensive clinical trials.

**Figure 2:**
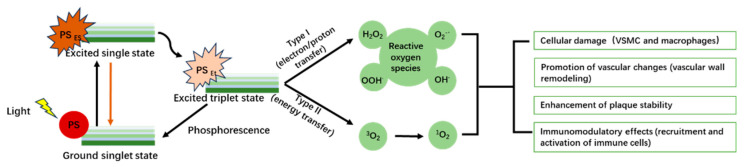
The mechanism of PDT in atherosclerosis.

The therapeutic effects of ROS generated during PDT in the treatment of atherosclerosis include direct cellular damage, promotion of vascular changes, and enhancement of plaque stability [88] (Figure 2). ROS can induce apoptosis and necrosis in various cellular components of plaques, such as vascular smooth muscle cells and macrophages, thereby reducing the cellular elements that contribute to plaque stability and progression [89]. Additionally, ROS contribute to vascular wall remodeling by inducing changes that can lead to the regression or stabilization of atherosclerotic plaques, including modulation of genes involved in inflammation and cellular adhesion, which are crucial in the pathology of atherosclerosis. By decreasing the macrophage content and affecting the fibrous cap, PDT can increase plaque stability, reducing the risk of rupture, which is a significant cause of acute vascular events such as myocardial infarction [90]. The precise control of light delivery, depth of penetration, and the specific activation of the photosensitizer enable PDT to target plaques effectively while preserving the integrity of the vascular wall, minimizing systemic effects, and focusing therapeutic actions at the site of disease, thus enhancing the safety and efficacy of the treatment for atherosclerosis [91]. Ongoing research efforts aim to optimize photosensitizer properties, improve light delivery techniques, and refine treatment protocols to maximize therapeutic outcomes while minimizing adverse effects [13,92].

The immunomodulatory effects of PDT on atherosclerosis are multifaceted and complex [88]. Following treatment, there is a rapid influx of neutrophils to the site, triggered by oxidative stress and cellular damage, which contribute to the acute inflammatory response and help clear cellular debris. PDT also induces apoptosis in macrophages, reducing the inflammatory cell burden within the plaque, and may shift their phenotype from pro-inflammatory (M1) to reparative (M2), promoting inflammation resolution and tissue healing [93]. Dendritic cells, critical for antigen presentation and immune regulation, can be affected by the altered cellular environment post-PDT, potentially impacting their maturation and ability to activate T-cells, with downstream effects on immune tolerance or activation. Furthermore, PDT stimulates the release of cytokines and chemokines that can either enhance inflammation resolution or contribute to a more chronic inflammatory state, depending on the balance of signals post-treatment. The induction of immunogenic cell death by PDT may also release damage-associated molecular patterns (DAMPs), which can either promote healing or exacerbate local inflammation [94,95]. In conclusion, the immunomodulatory effects of PDT in atherosclerosis are a result of the therapy’s induction of oxidative stress and subsequent cellular responses, influencing various immune pathways and cell types.

### 3.1. Early Discoveries in PDT for Atherosclerosis

Exploring the foundational efforts in PDT for atherosclerosis, early investigations have shed light on the potential mechanisms and efficacy of using specific lasers to regress hypertrophic intima, marking the initial steps towards non-invasive treatment alternatives. In 1999, Amemiya et al.’s study examined the efficacy of PDT using an Yttrium aluminum Garnet-optical parametric oscillator (YAG-OPO) laser for the treatment of atherosclerosis in rabbits, in comparison to the effects of PDT using an Argon-dye (Ar-dye) laser [96]. The study induced atherosclerosis in rabbits via a combination of balloon denudation injury and a subsequent high-cholesterol diet, followed by PDT after administration of the photosensitizer Photofrin. The results demonstrated a marked regression of hypertrophic intima and media in the arterial wall, without inducing inflammation, in YAG-OPO laser-treated arteries. Specifically, the intimal to medial thickness ratio (I/M ratio), a key indicator of arterial lesion regression, showed a significant reduction to 1.08 ± 0.54 two weeks post YAG-OPO laser treatment from a baseline equivalent to control groups around 3.69 ± 0.25. Importantly, PDT with YAG-OPO laser was markedly more effective in reducing arterial wall hypertrophy than with continuous-wave Ar-dye laser treatment, as evidenced by greater lumen enlargement and a less inflammatory response. This suggests that the pulse wave approach of the YAG-OPO laser not only effectively targets the intima but also the deeper medial layer without the thermal damage associated with continuous wave lasers. The study’s significance lies in its demonstration of PDT’s potential for non-inflammatory regression of atherosclerotic lesions, offering an innovative approach towards the challenging problem of restenosis post-angioplasty. However, the study’s limitation centers on its short-term observation period and the need for investigation into long-term outcomes, particularly concerning the potential for arterial wall weakening due to the extensive regression of both intima and media.

### 3.2. Innovative Optical Delivery Systems

As PDT evolves, significant advancements have been made in developing innovative delivery systems designed to enhance the precision, efficiency, and uniformity of light exposure across complex tissue structures, revolutionizing the application of PDT in clinical settings. Dwyer et al. present a study introducing an innovative balloon device designed for the application of PDT to anatomically complex tissue surfaces [97]. This optically integrated balloon utilizes the principle of multiple internal diffuse reflections to attain uniform illumination across various organ shapes. Constructed from medical-grade silicone incorporating titanium dioxide particles to enhance optical scattering, the device demonstrated impressive optical properties, with wall diffuse reflectance rates between 80 and 95%, and efficiencies typically around 65%. Efficiency improved with the balloon’s inflation, maintaining nearly constant irradiance despite changes in volume, signifying a notable advance in light delivery for PDT. This advancement is particularly significant for treating surfaces that are not easily accessible or conformable with traditional PDT devices, such as the oral mucosa and uterine endometrium, where the device has already seen clinical application. The key novelty of the work lies in its ability to offer consistent and efficient light exposure over anatomically irregular surfaces, a considerable challenge with existing PDT methods. The significance of this innovation extends beyond the device’s immediate application to photodynamic therapy, potentially impacting a wide range of treatments involving light-sensitive drugs. However, while the device has demonstrated high efficiency and uniformity in light delivery, a potential limitation lies in its dependency on precise inflation for optimal performance, with the overall efficiency and uniformity slightly decreasing when the device is overly inflated due to the thinning of the balloon wall material. This factor could necessitate careful volume control during clinical procedures to ensure the best therapeutic outcome.

Waksman et al. conducted a comprehensive preclinical study to evaluate the efficacy of PhotoPoint PDT in stabilizing atherosclerotic plaques and preventing plaque progression in a New Zealand white rabbit model [98]. The study utilized 40 rabbits fed with a cholesterol diet and subjected to iliac artery balloon denudation, followed by randomized treatment with PDT (photosensitizer and light), photosensitizer alone, or light alone, evaluated at 7- and 28-days post-treatment. The results showed a significant reduction in plaque progression of approximately 35% at 7 days and 53% at 28 days post-PDT treatment compared to controls, alongside an increase in lumen patency. PDT treatment led to a dramatic decrease in the percentage of plaque area occupied by macrophages by approximately 98% at 7 days and sustained at approximately 92% at 28 days, and an increase in non-proliferating smooth muscle cells (SMCs) by approximately 220% at 28 days. Importantly, there was no evidence of negative arterial remodeling, thrombosis, or aneurysm formation. This study illustrates the potential of PDT as a promising intervention for the treatment of acute coronary syndromes by substantially reducing plaque inflammation and favorably altering plaque composition towards a more stable SMC-rich phenotype, which is crucial for reducing disease progression. The novelty of this work lies in the dual effect of PDT in simultaneously reducing plaque inflammation and encouraging plaque stabilization through SMC repopulation without adverse arterial remodeling. The major limitation of this study is its preclinical nature, limiting its immediate translation to human application without further studies to confirm its safety and efficacy in humans.

**Figure 3:**
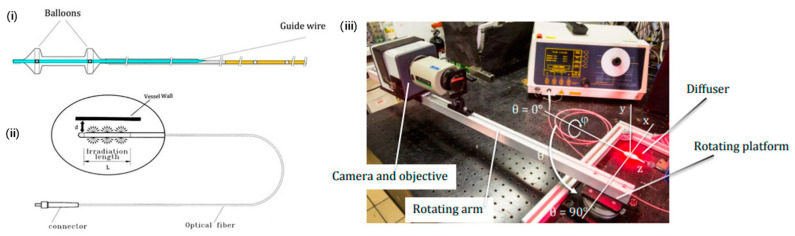
Innovative optical delivery system for intravascular PDT device. (**i**) Generic drawing of a stop-flow double balloon, multi-lumen nylon catheter; (**ii**) generic drawing of radial/cylindrical light diffuser for endovascular illumination; (**iii**) goniometer setup: rotating platform, mechanical arm with EM-CDD camera, neutral density filter and diffuse holder. Ref. [99], Copyright 2020, MDPI.

Zellweger et al.’s study offers a comprehensive exploration of the optical characteristics of an intravascular PDT device aimed at addressing vascular pathologies, notably atherosclerotic plaque [99]. The research delves into the intricacies of delivering light through a catheter and diffuser system explicitly developed for intra-arterial applications (Figure 3). Utilizing a planar imaging goniometer, they measured the radiance profiles longitudinally and angularly at 652 nm wavelength, revealing that the system emits near-Lambertian and “top hat” profiles. This discovery is pivotal for establishing accurate light dosimetry, a critical factor in ensuring the efficacy of PDT treatments. The study articulates that such an illumination profile allows for minimal deviations when the diffuser is placed within a catheter sheath, indicating an efficacious light delivery mechanism for vascular applications without significantly altering its optical output. The novel findings underscore the potential to optimize PDT treatments for atherosclerosis by enabling uniform light distribution within the arterial system, thus maximizing therapeutic outcomes while mitigating adverse effects. However, the exploration acknowledges a limitation in its scope by not thoroughly investigating the variability in optical properties due to different vascular geometries or tissue types. This highlight suggests avenues for future research to further refine the PDT approach for vascular applications.

### 3.3. Innovative Photosensitizers

Recent research has focused on elucidating the mechanisms of action and enhancing the targeting capabilities of PDT for atherosclerosis, demonstrating the therapy’s versatility in addressing the underlying pathophysiology of plaque development and progression through innovative therapeutic strategies. Hayase et al. investigated the effectiveness of photoangioplasty using locally delivered motexafin lutetium (Lu-Tex) in reducing macrophages and plaque burden in a rabbit model post-balloon injury, leveraged for its potential in the treatment of atherosclerosis [90]. Utilizing a high-cholesterol diet and balloon denudation to induce bilateral iliac artery lesions in 17 rabbits, the study uniquely focused on the direct intra-arterial delivery of Lu-Tex, followed by photoactivation (180 J/cm fiber at 200 mW/cm fiber). Local delivery led to immediate high concentrations of Lu-Tex within the plaque, achieving selective localization with mean concentrations notably higher in treated areas compared to untreated (14.02 ± 8.3 mg/g in treated iliac arteries vs. 0.61 ± 0.13 mg/g in untreated). This targeted approach resulted in a significant reduction of macrophage presence in treated lesions compared to untreated, particularly in both the intima (5% vs. 22%, *p* < 0.01) and media (8% vs. 23%, *p* < 0.01), with a modest yet significant decrease in plaque burden (73% vs. 82% plaque area, *p* < 0.01) without medial damage. The study demonstrates the feasibility and efficacy of localized Lu-Tex delivery and photoangioplasty in reducing macrophage density and plaque area in atherosclerotic lesions, emphasizing the therapeutic potential of photoangioplasty in atherosclerosis treatment by selectively targeting and reducing macrophage-rich atherosclerotic plaques without compromising the integrity of the normal vessel wall. However, the study’s limitation lies in the modality’s singular focus on macrophage reduction without examining the broader implications on overall plaque stability or regrowth over time.

Pai et al. embarked on a pioneering study to assess the potential of PDT, which combines light and a photosensitizing agent in the presence of oxygen, as a preemptive treatment for in-stent restenosis in rabbit iliac arteries, a condition that arises from the excessive proliferation of vascular smooth muscle cells post-angioplasty with stent deployment [100]. Utilizing 5-aminolaevulinic acid as the photosensitizing agent at a dose of 60 mg/kg, they illuminated the arteries at 635 nm with a total light dose of 50 J/cm^2^ either immediately before or after stent deployment. Early findings at three days post-treatment revealed nearly complete ablation of the medial cells when light was applied before stent deployment (approximately 17 ± 1 cells per high power field [hpf]), as opposed to the minimal effect when the illumination was post-stent deployment (approximately 184 ± 17 cells/hpf). At 28 days, neointimal areas were significantly reduced in the group treated with light before stent deployment (0.60 ± 0.21 mm^2^) compared to controls (1.41 ± 0.52 mm^2^) and the group with post-stent light application (1.24 ± 0.54 mm^2^), highlighting the method’s efficacy in inhibiting in-stent restenosis. This research propels PDT into consideration as a viable adjunct to percutaneous interventions in vascular diseases, especially given its safety profile, as evidenced by the lack of adverse events and maintenance of vessel patency throughout the study duration. The innovation lies in demonstrating PDT’s effectiveness in restenosis prevention, particularly when administered before stent placement, leveraging its cellular ablation capabilities. However, the limitation is the study’s focus on normal, non-atherosclerotic rabbit arteries, which may not fully replicate the complexity of human atherosclerotic vessels, thereby necessitating further investigation in clinically relevant models.

**Figure 4:**
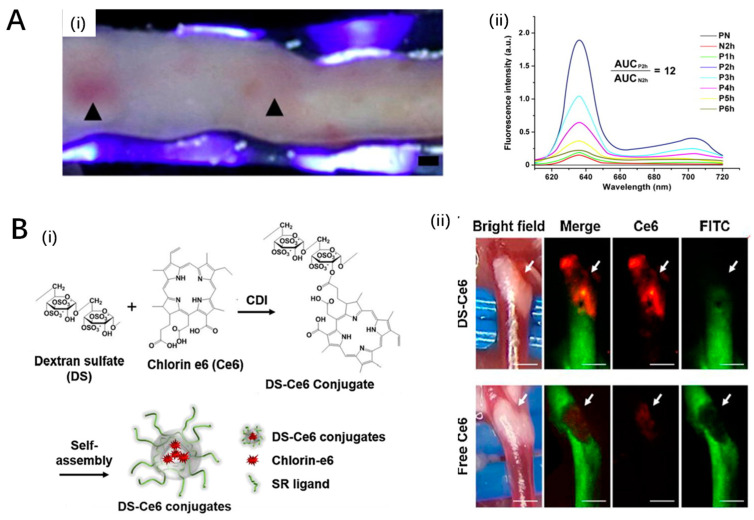
Innovative photosensitizers. (**A**) The fluorescence characteristics of PpIX in the plaque: (**i**) Excited by 405 nm light, the internal surface of the plaque exhibited a red fluorescence.; (**ii**) Fluorescence spectra were measured from plaques after ALA administration. Ref. [101], Copyright 2011, Elsevier. (**B**) A macrophage targetable near-infrared fluorescence emitting phototheranostic agent by conjugating dextran sulfate (DS) to chlorin e6 (Ce6): (**i**) chemical structure and synthetic procedure of DS-Ce6; (**ii**) in vivo imaging of the carotid atheroma at 48 h after the intravenous injection of DS-Ce6 or free Ce6, the result demonstrates the DS-Ce6 uptake in a carotid plaque. Ref. [103], Copyright 2021, BMC.

In the study conducted by Peng et al., the application of PDT utilizing 5-aminolevulinic acid (ALA)-derived protoporphyrin IX (PpIX) was investigated for the detection and treatment of inflamed atherosclerotic plaques in the carotid artery of rabbit models [101]. The study revealed that the peak fluorescence intensity of PpIX in plaques was observed 2 h after ALA injection, which was 12 times stronger than the adjacent normal vessel segment (Figure 4A). This fluorescence intensity showed a positive correlation with macrophage content (r = 0.794, *p* < 0.001), supporting the hypothesis that ALA-derived PpIX could reflect macrophage infiltration in plaques. The efficacy of PDT (635 nm at 50 J/cm^2^) was evaluated by comparing treated atherosclerotic plaques (n = 48) with control groups, demonstrating a 59% reduction in plaque area (*p* < 0.001) and significant decreases in macrophage content by 56% at 1 week and 64% at 4 weeks post-PDT. Additionally, collagen content in plaques increased by 44% at 4 weeks post-PDT (*p* < 0.05), suggesting that ALA-mediated PDT could not only inhibit plaque progression but also promote plaque stability by reducing macrophage content and enhancing collagen content. This study signifies a novel approach to treating inflamed atherosclerotic plaques by targeting macrophage content, an essential factor in plaque vulnerability, with ALA-mediated PDT. The use of ALA-derived PpIX as a fluorescence marker for macrophage content in plaques provides a promising method for both the detection and stabilization of vulnerable plaques, potentially reducing the risk of cardiovascular events. However, the major limitation of this work is its focus on an animal model, necessitating further studies to evaluate the safety, efficacy, and practical application of this therapy in human subjects.

In 2021, Wang et al. investigated the therapeutic potential of curcumin-mediated photodynamic therapy (CUR-PDT) on vascular smooth muscle cells (VSMCs) transformed by oxidized low-density lipoprotein (ox-LDL), focusing on autophagy’s role in inhibiting atherosclerosis (AS) progression [102]. Utilizing mouse aortic and A7r5 cell lines, they established an in vitro atherosclerosis model by treating VSMCs with ox-LDL, which induced a phenotypic transformation from a contractile to a synthetic phenotype, increased migration, and intracellular lipid accumulation. CUR-PDT significantly reversed these ox-LDL-induced alterations by promoting autophagy, as evidenced by the upregulation of LC3B-II and Beclin-1 and the downregulation of p62. Phenotypic markers α-SMA and SM22-α were upregulated, and osteopontin (OPN) was downregulated in CUR-PDT-treated cells. The migration ability and lipid droplet formation in VSMCs were considerably reduced post-CUR-PDT treatment. Notably, the inhibition of autophagy with 3-methyladenine reversed the protective effects of CUR-PDT, underscoring the central role of autophagy in CUR-PDT’s mechanism of action. This research highlights the potential of CUR-PDT as a novel therapeutic strategy against atherosclerosis by promoting autophagy in VSMCs. The study sheds light on an innovative application of CUR-PDT in cardiovascular diseases, particularly in atherosclerosis management, by modulating VSMC behavior. The major limitation of this study is its in vitro nature, calling for further in vivo studies to validate the findings and explore the specific molecular mechanisms and pathways involved in CUR-PDT-induced autophagy and its implications for atherosclerosis progression.

Song et al. (2021) investigated the feasibility of a macrophage-targeted phototheranostic agent for atherosclerosis, focusing on mechanisms by which dextran sulfate-conjugated chlorin e6 (DS-Ce6) influences atheroma through photoactivation [103]. The study found that DS-Ce6, via scavenger receptor-A mediated endocytosis, localized efficiently within activated macrophages and foam cells, and its photoactivation with light illumination mitigated atheroma burden and inflammation in murine models (Figure 4B). This effect was evidenced by a significant increase in macrophage-associated apoptotic bodies and the induction of autophagy, suggesting enhanced efferocytosis within atheroma. Importantly, photoactivation utilizing laser irradiation led to a notable reduction in plaque burden and inflammation by initiating apoptosis, autophagy, and subsequent lesional efferocytosis, paving the way for a promising theranostic strategy for high-risk atheroma. The innovative aspect of this research lies in the simultaneous detection of inflammatory activity and reduction of plaque burden in vivo through a targeted phototheranostic approach that demonstrated a harmonic contribution of apoptosis, autophagy, and efferocytosis towards mitigating atherosclerosis. However, the study’s significant limitation pertains to the single-disease model evaluation, suggesting the necessity for further research across diverse cardiovascular conditions to substantiate the findings and confirm replicability across a broader spectrum of atheromatous diseases.

## 4. Nanoparticle-Based PDT Approaches

The application of nanoparticles (NPs) in PDT represents a significant advancement [104,105,106,107,108]. This innovative approach leverages the unique properties of various nanoparticles to enhance the delivery and activation of photosensitizers (PSs), thus improving the efficacy and selectivity of PDT. Herein, we discuss the development and implications of polymeric nanoparticles, liposomal nanoparticles, upconversion nanoparticles (UCNPs), other inorganic nanoparticles, and protein nanoparticles (Table 2).

### 4.1. Polymeric Nanoparticles

Polymeric nanoparticles offer a versatile platform for PDT due to their biocompatibility, biodegradability, and ability to encapsulate both hydrophobic and hydrophilic PSs. The use of FDA-approved polymers such as PLGA (poly(lactic-co-glycolic acid)) enhances the clinical acceptability of these systems [109,110]. By modifying the polymer composition, molecular weight, and surface characteristics, polymeric nanoparticles can be tailored to control the release kinetics of PSs, target specific tumor tissues, and minimize side effects. Moreover, the surface functionalization of these nanoparticles allows for the conjugation of targeting ligands, enabling active targeting of cancer cells or the tumor microenvironment.

PDT represents an innovative approach for treating atherosclerosis by targeting atheromatous plaques with polyamidoamine (PAMAM) dendrimers or PAMAM/zinc phthalocyanine (ZnPc) conjugates, leveraging the advantages of PAMAM dendrimers as efficient drug carriers due to their low toxicity and improved drug solubility. Spyropoulos-Antonakakis et al. investigated the aggregation characteristics of zero-generation PAMAM dendrimers and their conjugates with ZnPc on human carotid tissues, employing atomic force microscopy (AFM) alongside statistical surface analysis and fractal analytical methodologies to evaluate texture characteristics [111]. A new conjugate of gallato zirconium (IV) phthalocyanine complexes (PcZrGallate) has been obtained from alkilamino-modified SiO_2_ nanocarriers (SiO_2_-(CH_2_)_3_-NH_2_ NPs), which may potentially be used in photodynamic therapy of atherosclerosis. Its structure and morphology have been investigated. The photochemical properties of the composite material have been characterized. in saline environments when exposed to different light sources. Reactive oxygen species (ROS) generation in DMSO suspension under near IR irradiation was evaluated. The PcZrGallate-SiO_2_ conjugate has been found to induce a cytotoxic effect on macrophages after IR irradiation, which does not correspond to ROS production. It was found that SiO_2_ as a carrier helps the photosensitizer enter the macrophages, a type of cell that plays a key role in the development of atheroma. These properties of the novel conjugate may make it useful in the photodynamic therapy of coronary artery disease. Their findings revealed a differential aggregation behavior of the nanocarriers and nanodrugs on symptomatic (atheromatous) versus asymptomatic (healthy) tissues; notably, larger aggregations of G0/ZnPc were observed on atheromatous plaques. This aggregation pattern underscores the potential of using PAMAM dendrimer carriers in PDT for atherosclerosis, where the target-selective necrosis of macrophage cells within atheromatous plaques is crucial. The study demonstrated not just the selective aggregation of these nanocarriers on diseased tissues but also underscored the complex interplay between nanothermodynamic conditions, aggregation, and drug delivery efficacy, revealing a novel platform for PDT in treating cardiovascular diseases. However, the work also highlights a significant limitation: the understanding of the precise mechanisms governing the intracellular translocation and selectivity of these nanocarriers remains limited, necessitating further investigation.

Wennink et al. investigated the potential of m-tetra(hydroxyphenyl)chlorin (mTHPC)-loaded polymeric micelles, based on benzyl-poly(ε-caprolactone)-b-methoxy poly(ethylene glycol) (Ben-PCL-mPEG), for macrophage-selective PDT as a novel treatment approach for atherosclerosis [112]. Utilizing a film hydration method, they achieved a high loading efficiency of 17% for mTHPC in the micelles. These micelles demonstrated higher lipase-mediated degradation and photosensitizer release in RAW264.7 macrophages compared to C166 endothelial cells, indicating cell-type selective photocytotoxicity in vitro. Interestingly, in vitro stability tests revealed rapid release of mTHPC from the micelles within 30 min in blood plasma, suggesting a similar in vivo pharmacokinetics behavior between the micellar formulation and free mTHPC. Despite this, mTHPC accumulation was observed in atherosclerotic lesions in mouse models, likely due to lipoprotein binding post-release. The study suggests the potential of mTHPC-loaded Ben-PCL-mPEG micelles in targeting macrophages within atherosclerotic plaques, highlighting the importance of enhancing stability to fully leverage macrophage selectivity. The rapid release of mTHPC in biological media poses a significant limitation, underlining the need for further formulation optimization to improve in vivo stability and selectivity.

### 4.2. Liposomal Nanoparticles

Liposomes, as biocompatible and biodegradable vesicles, have been extensively explored for PDT [113,114,115]. Their ability to encapsulate both hydrophilic PSs in the aqueous core and hydrophobic PSs within the lipid bilayer makes them highly versatile. Surface modification with polyethylene glycol (PEG) can extend their circulation time, enhancing tumor accumulation via the enhanced permeability and retention (EPR) effect. The incorporation of targeting moieties further allows for the selective delivery of PSs, improving the therapeutic index of PDT.

In a study by Jain et al., the potential of intra-arterial PDT using Visudyne^®^ (Liposomal Verteporfin) for the treatment of atherosclerotic plaque was systematically investigated [116]. This approach hypothesized that local intra-arterial delivery of the photosensitizer followed by photo-activation could result in rapid plaque accumulation and limited systemic side effects, thus aligning with percutaneous coronary intervention practices. Employing atherosclerotic aorta isolated from ApoE^−/−^ mice, the study characterized dose-dependent Visudyne^®^ uptake, showing significant fluorescence intensity increases correlating with rising doses of 100 (FI-5.5 ± 1.8), 200 (FI-31.9 ± 1.9), and 500 ng/mL (FI-42.9 ± 1.2), and administration times up to 15 min, achieving saturation by 30 min. Notably, post-Visudyne^®^ perfusion (500 ng/mL for 15 min) and PDT (at 100 and 200 J/cm^2^), marked increases in reactive oxygen species (ROS) and apoptotic cells, particularly macrophages, were observed, with minimal adverse effects on the medial wall, suggesting a targeted action of the therapy. Visudyne^®^-PDT notably induced macrophage apoptosis and attenuated smooth muscle functionality, suggesting a plausible therapeutic strategy for altering the progression of plaque development. This work indicates the feasibility of targeted intravascular administration of photosensitizers for plaque treatment, highlighting the specificity and effectiveness of Visudyne^®^-PDT in inducing apoptosis in plaque macrophages while posing potential limitations related to the applicability of different plaque compositions and vessel geometries within human subjects.

**Figure 5:**
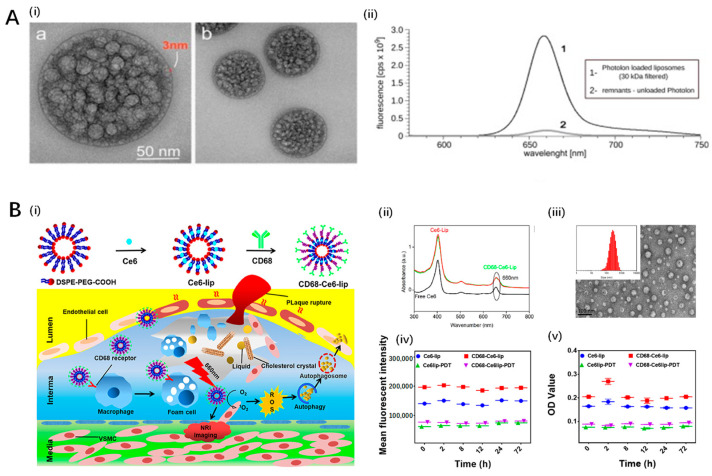
Liposome based nanoparticles for PDT in atherosclerosis. (**A**) a novel liposomal formulation of PVP-conjugated chlorin e6 (Photolon): (**i**) transmission electron micrographs of liposomal formulation of Photolon; (**ii**) fluorescence spectra indicating high encapsulation efficiency of Ce6 in nanoparticles. Ref. [117], Copyright 2019, MDPI. (**B**) CD68-modified Ce6-loaded liposomes for PDT in atherosclerosis: (**i**) schematic illustration of the preparation of CD68-Ce6-lip and their phototherapeutic mechanism at the atherosclerotic plaques; (**ii**) UV spectra of CD68-Ce6-lip demonstrates Ce6 was encapsulated in liposomal nanoparticles; (**iii**) TEM images and particle size distribution of Ce6-lip; (**iv**,**v**) the liposomes have good fluorescence stability and UV stability after 660 nm laser irradiation for 20 min. Ref. [118], Copyright 2023, Elsevier.

Jain et al. investigated the use of intra-arterial photodynamic therapy (PDT) with Visudyne^®^ for the treatment of atherosclerotic plaque. In contrast, Kałas et al. focused on a novel liposomal formulation of chlorin e6, highlighting the diverse approaches being explored for targeted plaque reduction. Kałas et al. developed a novel liposomal formulation of PVP-conjugated chlorin e6 (Photolon), achieving an encapsulation efficiency of 93 ± 6%, which facilitates the selective delivery of this photoactive drug for the photodynamic reduction of atherosclerotic plaque [117]. The findings, derived from both in vitro and in vivo models, reveal that the developed liposomes, characterized by an average diameter of 124.7 ± 0.6 nm and a polydispersity index (PDI) of 0.055, are effectively taken up by macrophages without cytotoxicity in the absence of light (Figure 5A). Notably, this formulation exhibited promising pharmacokinetics in Sus scrofa f. domestica, with a half-life of 20 min and an area under the curve (AUC) of 14.7, showing rapid clearance that aligns with the objectives of minimizing systemic toxicity. In vitro studies further underscored its potential in arteriosclerosis treatment by demonstrating selective cytotoxicity towards macrophages upon light activation, key agents in the development of atherosclerotic plaque, without harming vascular smooth muscle or endothelial cells. This selective accumulation and phototoxic response in macrophages highlight its innovativeness for targeted atherosclerosis therapy. However, a notable limitation is the unexplored long-term biocompatibility and potential immune response triggered by the liposomal formulation, which demands further investigation.

In addressing the challenge of targeted PDT for atherosclerosis, Zou et al. developed chlorin e6 (Ce6)-loaded, CD68-modified liposomes, leveraging the macrophage-targeting ability of CD68 antibodies to enhance therapeutic efficacy [118]. The encapsulation efficiency of Ce6 in the liposomes was around 68.7%, with a drug loading percentage of approximately 3.8%. In vitro studies demonstrated that these liposomes, upon laser irradiation, significantly inhibited the migration of mouse aortic vascular smooth muscle cells and induced cholesterol efflux and autophagy in macrophage-derived foam cells, pivotal factors in the development of atherosclerosis (Figure 5B). Specifically, the study found that the liposomes promoted autophagy through increased LC3-I and LC3-II expression while decreasing p62 expression, indicating enhanced autophagic flux. Crucially, the engineered liposomes did not exhibit significant cytotoxicity to human coronary artery endothelial cells under the examined conditions, suggesting a degree of biocompatibility. The leverage of CD68 targeting considerably enhanced the specific cellular uptake by macrophage-derived foam cells, providing a targeted therapeutic approach that could notably reduce the systemic side effects commonly associated with PDT. This study underscores the potential of CD68-Ce6-mediated liposomes as a promising nano-drug delivery system for atherosclerosis treatment, offering a viable alternative with enhanced specificity and efficacy. However, the in vivo therapeutic efficacy and safety of these liposomes remain to be thoroughly investigated, marking the primary limitation of this work.

### 4.3. Upconversion Nanoparticles

UCNPs represent a groundbreaking class of nanomaterials that can convert near-infrared (NIR) light to higher-energy visible light, enabling the activation of PSs deep within tissues where light penetration is limited [119,120,121,122]. This unique property opens new avenues for treating deep-seated tumors with PDT. The coupling of PSs to UCNPs requires careful consideration of the energy transfer efficiency, which is influenced by the proximity and spectral overlap between the UCNP emission and the PS absorption. Recent advancements include the development of UCNPs that exhibit optimized NIR absorption and minimal cytotoxicity, improving the safety and efficacy of UCNP-mediated PDT.

**Figure 6:**
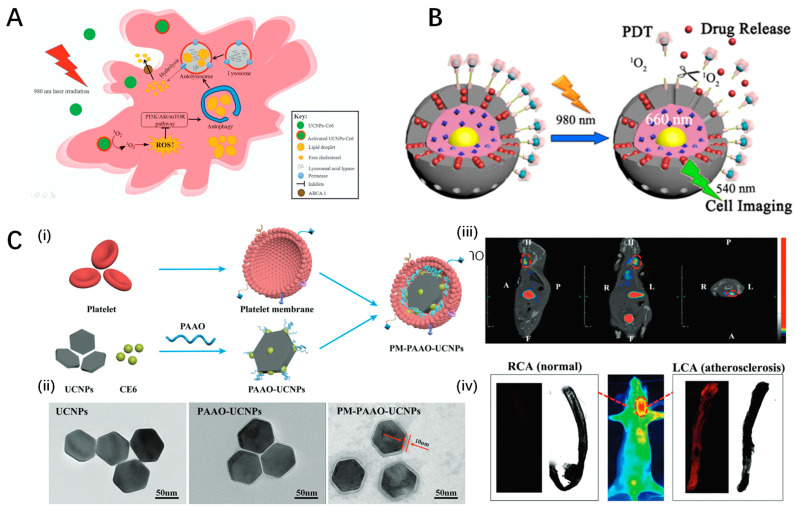
Upconversion nanoparticles for PDT in atherosclerosis. (**A**) Schematic diagram of UCNPs-Ce6-mediated PDT and the mechanism of autophagy. Ref. [123], Copyright 2017, Nature. (**B**) Core–shell–shell upconversion nanoparticles for NIR-Induced photodynamic therapy. Ref. [124], Copyright 2016, American Chemical Society. (**C**) Platelet membrane coated upconversion nanoparticles accumulate in atherosclerotic plaque: (**i**) schematic illustration showing the composition of PM-PAAO-UCNPs; (**ii**) TEM images of nanoparticles; (**iii**,**iv**) SPECT/CT and fluorescence images of PM-PAAO-UCNPsin model mouse illustrating nanoparticles accumulate in plaque. Ref. [125], Copyright 2021, Wiley-VCH GmbH.

Han et al. explored the efficacy of upconversion nanoparticle-mediated PDT using upconversion fluorescent nanoparticles encapsulating chlorin e6 (UCNPs-Ce6) in promoting cholesterol efflux from macrophage-derived foam cells, crucial in atherosclerotic plaque development [123,124]. This study initially observed that PDT significantly increased cholesterol efflux and induced autophagy in THP-1 and peritoneal macrophage-derived foam cells (Figure 6A,B). Autophagy inhibition by 3-methyladenine or ATG5 siRNA reduced PDT-induced autophagy and ABCA1-mediated cholesterol efflux, highlighting autophagy’s central role in this process. Reactive oxygen species (ROS) generated by PDT were identified as the primary inducers of autophagy, with N-acetyl cysteine (NAC) reversing these effects. Furthermore, PDT’s influence on the ROS/PI3K/Akt/mTOR signaling pathway underscored its mechanism of action. Notably, this study affirms the potential of UCNPs-Ce6-mediated PDT in enhancing cholesterol efflux through autophagy induction and ROS generation, offering a novel therapeutic strategy against atherosclerosis progression. The approach’s limitation lies in its dependency on ideal PDT conditions and the complexity of in vivo applications, necessitating further exploration to validate effectiveness and safety in clinical settings.

Building upon the therapeutic potential of upconversion nanoparticle-mediated photodynamic therapy, Ma et al. introduced a platelet-mimicking photodynamic therapeutic system designed to target and mitigate the progression of atherosclerotic plaques noninvasively, aimed at overcoming limitations faced by conventional clinical strategies such as surgery and long-term drug administration [125]. This innovative approach utilizes photosensitizer-loaded upconversion nanoparticle cores coated with platelet membrane to specifically target macrophage-derived foam cells, a key component in early-stage atherosclerotic plaques (Figure 6C). In vivo studies highlighted the system’s effectiveness in plaque targeting and treatment, as confirmed by in vivo fluorescent and single-photon emission computed tomography/computed tomography (SPECT/CT) radionuclide imaging. This targeted delivery and treatment led to foam cell apoptosis and reduced inflammation, suggesting a promising noninvasive strategy for addressing atherosclerosis progression. The platelet-membrane coating significantly prolongs plasma retention times, enhancing the system’s efficiency in targeting and treating atherosclerotic sites. However, the potential long-term impact and the specificity of the system’s plaque targeting require further investigation to fully understand its limitations and applicability in clinical settings.

Another innovative strategy for harnessing photodynamic therapy for atherosclerosis treatment involves the use of a protein-polysaccharide nanoemulsion co-loaded with chlorin e6 and upconversion nanoparticles. In a study by Huang et al., a novel approach for atherosclerosis (AS) treatment involving a protein–polysaccharide nanoemulsion co-loaded with a photosensitizer (chlorin e6, Ce6) and upconversion nanoparticles (UCNPs) (UCNPs-Ce6@DB) was developed, targeting macrophage-derived foam cells in AS plaques [126]. The nanoemulsion was designed to enhance the delivery and efficacy of PDT by utilizing UCNPs to convert near-infrared light to the UV–Vis region, activating Ce6 for ROS generation, which in turn induces macrophage autophagy. This process resulted in improved lipid metabolism and reduced pro-inflammatory cytokine secretion in vitro. The nanoemulsion showed preferential uptake by macrophage-derived foam cells via scavenger receptor class A (SR-A)-mediated endocytosis and demonstrated a significant reduction in plaque formation in ApoE^−/−^ mice under NIR irradiation. The treatment increased the expression of ABCA1, a transporter involved in cholesterol efflux, and decreased levels of TNF-α, IL-1β, and IL-6, indicating a potent anti-inflammatory effect. The in vivo experiments also showed a considerable reduction in necrotic cell areas and lipid plaques on the vascular surface of treated mice, highlighting UCNPs-Ce6@DB’s potential as a promising AS treatment through PDT. This study’s novelty lies in the innovative combination of UCNPs and Ce6 in a protein–polysaccharide nanoemulsion, enabling targeted, efficient treatment of AS by leveraging PDT-induced autophagy. However, the major limitation of this work is the reliance on NIR irradiation for PDT, which may limit the treatment’s applicability to lesions accessible by such irradiation methods.

### 4.4. Other Inorganic Nanoparticles

Various inorganic nanoparticles, including gold nanoparticles and quantum dots, have been investigated for their potential in PDT. These nanoparticles can act as PS carriers or as energy transducers. Gold nanoparticles, particularly gold nanorods, harness plasmonic properties to enhance local light absorption and can be engineered to convert NIR light to heat, offering a synergistic photothermal effect alongside PDT. Quantum dots, with their size-tunable optical properties, serve as efficient energy donors in FRET-based systems to activate attached PSs.

Gerasymchuk et al. developed a novel conjugate for PDT, specifically targeting macrophages involved in atherosclerotic plaque development [127]. The conjugate is a gallato zirconium (IV) phthalocyanine complex (PcZrGallate) bound to SiO_2_ nanocarriers, designed to enhance the delivery and efficacy of PDT in atherosclerosis treatment. Characterization revealed that this conjugate exhibits enhanced emission yield and significant reactive oxygen species (ROS) generation upon IR irradiation in both DMSO and saline environments, indicating its potential as a photoactive drug. Intriguingly, in vitro tests demonstrated that PcZrGallate–SiO_2_ conjugates possess lower dark cytotoxicity compared to the soluble form of PcZrGallate and can effectively sensitize RAW264.7 cells to IR light, marking a significant step towards developing more efficient PDT drugs. This research introduces a promising avenue for the treatment of unstable atherosclerotic plaques by harnessing the phototoxic effects of the newly developed conjugates, which combine the photodynamic capabilities of PcZrGallate with the cell-targeting proficiency of SiO_2_ nanocarriers. The approach’s novelty lies in the synthesis technique and the conjugate’s ability to interact with macrophages, offering a targeted PDT method for atherosclerosis without inducing undue toxicity in the absence of irradiation. However, the major limitation lies in the scalability of synthesis and the detailed in vivo toxicology and efficacy studies required to establish clinical relevance fully.

**Figure 7:**
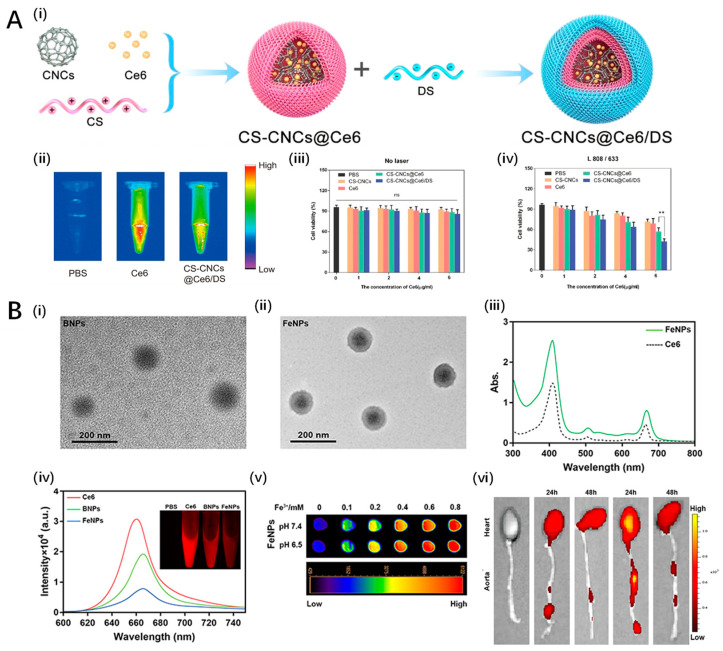
Other inorganic nanoparticles for PDT in atherosclerosis. (**A**) SR-A-targeted nanoplatform for sequential photothermal/photodynamic ablation of atherosclerosis (CS-CNCs@ Ce6/DS): (**i**) the key steps involved in the preparation of CS-CNCs@ Ce6/DS; (**ii**) high fluorescence signal was detected in nanoplatform; (**iii**,**iv**) CS-CNCs@ Ce6/DS nanoparticles have good irradiation toxicity in macrophages after irradiation (**iv**) compared with no laser irradiation (**iii**). Ref. [128], American Chemical Society. (**B**) A combination of MRI and chemiexcited PDT for atherosclerosis based on Fe^3+^: (**i**,**ii**) TEM images of nanoparticles (125.3 ± 6.4 nm); (**iii**) absorption spectra of FeNPs indicate Ce6 was encapsulated in FeNPs; (**iv**) fluorescence spectra of FeNPs indicate Ce6 was encapsulated in FeNPs; (**v**) T1-weighted MRI images of FeNPs at various concentrations; (**vi**) FeCNPs accumulate in aortae of high fat-diet ApoE^–/–^ mice. Ref. [131], Copyright 2022, Dove press.

Another study explored a targeted nanoplatform combining photothermal and photodynamic therapy to ablate activated macrophages, key contributors to atherosclerosis, while inhibiting oxidized low-density lipoprotein uptake and inflammatory processes. In a pioneering study by Liu et al., the highly targeted nanoplatform CS-CNCs@Ce6/DS was developed for sequential photothermal and photodynamic therapy (PTT/PDT) aimed at ablating activated macrophages in atherosclerotic plaques [128]. The innovative nanoplatform exploits chitosan-coated carbon nanocages (CS-CNCs) for loading chlorin e6 (Ce6) and incorporates dextran sulfate (DS) for specific targeting of the scavenger receptor type A (SR-A) on activated macrophages, which are key contributors to atherosclerosis (AS) development (Figure 7A). The study demonstrated the highly selective accumulation of CS-CNCs@Ce6/DS in activated macrophages, facilitating effective ablation via sequential PTT/PDT. DS not only provided targeting specificity but also competitively inhibited cellular endocytosis of oxidized low-density lipoproteins, mitigating AS progression. The sequential therapy, beginning with PTT followed by PDT, was found to enhance the efficiency of macrophage ablation due to a near-infrared-accelerated release of Ce6, leading to significant shrinkage and stabilization of atherosclerotic plaques in vitro and in ApoE−/− mouse models. The nanoplatform ensured a profound reduction in pro-inflammatory cytokine secretion and impeded the migration and proliferation of smooth muscle cells, crucial for plaque development. This study establishes a ‘greater than the sum of its parts’ effect for AS treatment, offering a promising avenue for preventing AS-related diseases. This research sets a novel paradigm in AS management by harnessing the synergy between PTT and PDT, directly targeting the inflammatory roots of atherosclerosis. A remarkable aspect of this work is its successful targeting and elimination of activated macrophages within atherosclerotic plaques, addressing the challenge of non-specificity in traditional AS therapies. However, the potential limitations of laser treatments, especially for coronary artery applications due to tissue penetration constraints, warrant further exploration to extend the platform’s clinical applicability.

Another innovative approach targeted activated macrophages in atherosclerotic plaques but employed a distinct nanoplatform and therapeutic modality. Cao et al. reported the development of copper sulfide/titanium oxide heterostructure nanosheets modified with hyaluronic acid and PEG (HA-HNSs) for macrophage-targeted sonodynamic and photothermal synergistic therapy against early atherosclerotic plaques [129]. Leveraging the high electron-hole separation efficiency and superior sonodynamic performance of the CuS/TiO_2_ heterostructure, along with the intense absorbance in the NIR-II region ensuring excellent photothermal performance, HA-HNSs were shown to selectively target and induce apoptosis in proinflammatory macrophages, which are pivotal in atherosclerotic plaque progression. In vivo and in vitro studies revealed that the combination of sonodynamic and photothermal therapy based on HA-HNSs effectively prevented the development of early atherosclerotic plaques by eliminating lesional macrophages and reducing inflammation. Specifically, the synergy between sonodynamic therapy (SDT) and photothermal therapy (PTT) was highlighted by a significantly reduced viability of macrophages treated with combined therapy, in comparison to each therapy applied separately. The encapsulation of HA ensured targeted delivery of the therapy, capitalizing on the CD44-HA interaction to effectively home the nanosheets to proinflammatory macrophages within plaques. This study emphasizes the novelty and potential clinical translational value of CuS/TiO_2_ nanosheets for noninvasive, targeted, and efficient treatment of early-stage atherosclerosis. A limitation of the work is the complexity of fully understanding and controlling the biodistribution of the nanosheets and the long-term effects of such treatments on the body’s macrophage population and broader immune responses.

Mu et al.’s innovative approach, which integrates chemiexcited photodynamic therapy with magnetic resonance imaging capabilities, signifies a substantial advancement in the evolving field of nanomedicine, synergistically combining diagnostic imaging and targeted treatment strategies to facilitate the development of precision medicine techniques for the treatment of atherosclerosis. Mu et al. innovatively developed chemiexcited PDT using polymeric nanoparticles integrated with magnetic resonance imaging (MRI) capabilities for atherosclerosis treatment, marking a strategic advancement in non-invasive cardiovascular therapies [130]. The system, designated as FeCNPs, incorporates chemical fuel (CPPO) and photosensitizers within a polymeric matrix that is cross-linked with Fe^3+^–catechol complexes, simultaneously enabling stabilization, targeted therapy, and imaging (Figure 7B). The FeCNPs were shown to preferentially accumulate in atherosclerotic plaques, as evidenced by persistent and enhanced T1-weighted MRI contrast, and effected macrophage elimination in plaques, reducing plaque size and thickness. This therapeutic action was quantitatively supported by the significant reductions in the expression levels of macrophage markers (CD68) and pro-inflammatory cytokines (MCP1 and TNFα) in treated plaques. Intriguingly, lower doses of FeCNPs demonstrated superior therapeutic effectiveness compared to higher doses, suggesting an optimal therapeutic window. However, the efficacy varied across different arterial regions, indicating the influence of plaque characteristics on therapeutic outcomes. This study is the first to report the integration of MRI imaging and chemiexcited PDT in a single system for the management of atherosclerosis, offering a promising route for the development of multifunctional nanotherapies. The key significance of this work lies in its potential to provide a non-invasive, targeted, and imaging-guided therapeutic approach for atherosclerosis—a leading cause of cardiovascular diseases. One major limitation is the non-uniform therapeutic efficacy across different aortic regions, which necessitates further investigations into targeted delivery optimizations.

### 4.5. Protein-Based Nanoparticles

Nature-derived protein nanoparticles, such as those based on albumin or gelatin, provide biocompatible and biodegradable options for PS delivery. These protein-based systems can be easily functionalized with targeting ligands and loaded with PSs through physical adsorption or covalent binding. Their inherent biocompatibility and potential for active targeting make them attractive candidates for clinical translation.

Banerjee et al. developed and evaluated zinc phthalocyanine-loaded human serum albumin nanoparticles (HSA NPs) for their application in PDT targeted at atherosclerotic plaques. Characterization of these nanoparticles demonstrated an average size of 120 nm, good loading capacity for zinc phthalocyanine, and stability in 50% fetal bovine serum for 8 h, as well as in aqueous conditions at 4 °C for 4–6 weeks. Under near-infrared (NIR) light irradiation, the nanoparticles produced a high yield of singlet oxygen without exhibiting dark toxicity toward macrophages in vitro [131]. Upon LED illumination, these nanoparticles efficiently induced macrophage cell death at low µg/mL concentrations. In a murine model of atherosclerosis, injection of these nanoparticles led to visible fluorescence signals from zinc phthalocyanine in the plaques and lung tissues within 30 min post-injection, with fast clearance observed thereafter. The study underscored the potential of zinc phthalocyanine-loaded HSA NPs as viable candidates for both the theranostic visualization and photodynamic treatment of macrophages within atherosclerotic plaques, demonstrating significant implications for advancements in cardiovascular disease treatment methods. Despite these encouraging results, a notable limitation involves the intricate balance between nanoparticle stability and effective release of the photosensitizer under physiological conditions. This work highlights an innovative approach towards localized treatment of atherosclerotic plaques, offering a dual-functional nanoparticle system with potential for further optimization and clinical exploration in cardiovascular therapeutics.

**Table 2:** The list of nanoparticle-based PDT used in treatment of atherosclerosis.

Study	Nanoparticle Approach	Brief Description
Spyropoulos-Antonakakis et al. 2015 [111]	Polymeric Nanoparticles	PAMAM dendrimers or PAMAM/zinc phthalocyanine (ZnPc) conjugates
Wennink et al. 2017 [112]	Polymeric Nanoparticles	m-tetra(hydroxyphenyl)chlorin (mTHPC)-loaded polymeric micelles based on benzyl-poly(ε-caprolactone)-b-methoxy poly(ethylene glycol) (Ben-PCL-mPEG)
Jain et al. 2016 [116]	Liposomal Nanoparticles	Visudyne^®^ (Liposomal Verteporfin)
Kałas et al. 2019 [117]	Liposomal Nanoparticles	Novel liposomal formulation of PVP-conjugated chlorin e6 (Photolon)
Zou et al. 2023 [118]	Liposomal Nanoparticles	Chlorin e6 (Ce6)-loaded, CD68-modified liposomes
Han et al. 2017 [123]	Upconversion Nanoparticles	Upconversion fluorescent nanoparticles encapsulating chlorin e6 (UCNPs-Ce6)
Ma et al. 2021 [125]	Upconversion Nanoparticles	Platelet-mimicking photodynamic therapeutic system using photosensitizer-loaded upconversion nanoparticle cores coated with platelet membrane
Huang et al. 2023 [126]	Upconversion Nanoparticles	Protein-polysaccharide nanoemulsion co-loaded with chlorin e6 and upconversion nanoparticles (UCNPs-Ce6@DB)
Gerasymchuk et al. 2021 [127]	Other Inorganic Nanoparticles	Gallato zirconium (IV) phthalocyanine complex (PcZrGallate) bound to SiO_2_ nanocarriers
Liu et al. 2021 [128]	Other Inorganic Nanoparticles	Highly targeted nanoplatform CS-CNCs@Ce6/DS using chitosan-coated carbon nanocages (CS-CNCs) loaded with Chlorin e6 (Ce6) and dextran sulfate (DS)
Cao et al. 2022 [129]	Other Inorganic Nanoparticles	Copper sulfide/titanium oxide heterostructure nanosheets modified with hyaluronic acid and PEG (HA-HNSs)
Mu et al. 2022 [130]	Other Inorganic Nanoparticles	Chemiexcited PDT using polymeric nanoparticles integrated with magnetic resonance imaging (MRI) capabilities (FeCNPs)
Banerjee et al. 2019 [131]	Protein-based Nanoparticles	Zinc phthalocyanine-loaded human serum albumin nanoparticles (HSA NPs)

## 5. Clinical Trials

With the advent of advanced imaging techniques, there is a burgeoning interest in identifying high-risk atherosclerotic plaques for targeted intervention, potentially revolutionizing predictive accuracy and preventive strategies in coronary artery disease management through PDT. The PROSPECT II study, led by David Erlinge and colleagues, aimed to investigate whether a combined near-infrared spectroscopy (NIRS) and intravascular ultrasound imaging approach could identify high-risk coronary artery plaques and patients at elevated risk for future major adverse cardiac events (MACEs) [132]. In a study conducted across multiple centers in Denmark, Norway, and Sweden, 898 patients with recent myocardial infarction were enrolled and underwent three-vessel coronary imaging post-treatment of obstructive lesions. Non-obstructive lesions were characterized using imaging modalities to assess lipid content and plaque burden. The study found that high lipid-rich plaques, identified in 59% of patients, significantly predicted patient- and lesion-level non-culprit lesion-related MACEs with adjusted odds ratios of 2.27 and 7.83, respectively. Specifically, lesions with both high lipid content and large plaque burden showcased the greatest risk for future MACEs, emphasizing the utility of combined NIRS and intravascular ultrasound in pinpointing vulnerable plaques. This comprehensive approach suggests a promising avenue for targeting preventive strategies in coronary artery disease management. The study was supported by Abbott Vascular (Chicago, IL, USA), Infraredx (Bedford, MA, USA), and The Medicines Company (Parsippany-Troy Hills, NJ, USA), highlighting a significant step toward integrating advanced imaging techniques in clinical practice for assessing coronary plaque vulnerability.

Clinical trials on PDT for treating atherosclerosis have also explored various photosensitizers and delivery methodologies to target and regress atherosclerotic plaques effectively [92]. These investigations aim to utilize the photochemical properties of specific agents to initiate cellular processes that minimize plaque burden and improve arterial health. Early studies with hematoporphyrin derivative (HpD) and its refined form, Photofrin, highlighted the potential of these agents in vascular PDT [96,133,134,135]. Spears et al. demonstrated selective accumulation of HpD within atherosclerotic plaques [136,137,138]. Clinical trials using Photofrin showed promising results in reducing restenosis post-angioplasty by causing apoptosis in vascular smooth muscle cells (VSMCs) without significantly damaging the surrounding tissues or without inducing in-stent restenosis [139,140,141]. Verteporfin, known for its applications in ophthalmology, was also studied for atherosclerosis. It efficiently targets macrophages within plaques due to enhanced uptake facilitated by binding to low-density lipoproteins. Light activation of verteporfin was found to promote plaque stability and reduce macrophage-induced inflammation, highlighting its potential as a vascular therapeutic agent. Studies with indocyanine green have explored its dual imaging and therapeutic capabilities in PDT. Troels et al. utilized ICG in a clinical setting where it was shown to reduce arterial wall thickness upon activation with near-infrared light, illustrating its beneficial role in targeting vascular smooth muscle cells and reducing neointimal hyperplasia.

5-aminolevulinic acid (ALA)-induced protoporphyrin IX is a significant focus of PDT research due to its proclivity to concentrate in atheromas after systemic administration [142,143]. The ease of application and activation using external light sources makes it particularly attractive for clinical use. Clinical trials demonstrated that ALA-based PDT could effectively prevent restenosis by reducing vascular smooth muscle proliferation and encouraging apoptotic pathways [101,144]. Motexafin Lutetium (Lutex), a third-generation photosensitizer, showed potential in phase I and phase II trials for reducing plaque burden [145]. It targets atheromas with high selectivity, and its activation leads to oxidative damage restricted to the targeted plaque areas, minimizing damage to healthy vascular structures. As an extension of PDT, sonodynamic therapy (SDT) uses ultrasonic waves instead of light to activate the photosensitizers. Trials with agents such as sinoporphyrin demonstrated that SDT could effectively reduce markers of vascular inflammation and stabilize plaques, offering a novel, non-invasive treatment modality for atherosclerosis.

## 6. Conclusions and Outlook

PDT offers a promising approach to the treatment of atherosclerosis, a predominant cardiovascular disease characterized by the accumulation of lipids, pro-inflammatory processes, and plaque formation in arterial walls. This innovative treatment modality leverages photosensitizers that, upon activation by specific light wavelengths, generate ROS capable of inducing cell death, modulating inflammatory responses, and stabilizing atherosclerotic plaques. The selective targeting capability of PDT, aiming primarily at atherosclerotic lesions while sparing healthy tissues, emerges as its key advantage, allowing for localized treatment with minimal systemic effects. Despite its potential, the clinical translation of PDT in atherosclerosis treatment entails overcoming significant challenges and limitations.

The application of PDT to atherosclerosis faces several limitations that warrant careful consideration for its advancement and integration into clinical practice. First, the limited penetration depth of light through vascular tissues presents a fundamental challenge, especially for atherosclerotic plaques located in deeper or more complex vascular regions. Enhancing light delivery systems, perhaps through the development of fiber-optic catheters or advancements in light technology, could improve PDT’s applicability and effectiveness. Second, achieving optimal photosensitizer specificity and concentration constitutes another critical aspect. The ideal photosensitizer for atherosclerosis treatment should exhibit high plaque selectivity to minimize effects on surrounding healthy tissues and ensure efficient ROS generation upon light activation. Developing novel photosensitizers or modifying existing ones to enhance their plaque-binding affinity and pharmacokinetic properties remains an area of active research. Third, the potential for collateral damage due to non-specific ROS generation necessitates precise control over the PDT process. While PDT aims to minimize systemic side effects, the risk of damaging adjacent healthy tissues highlights the importance of refining treatment protocols, including light dosage and exposure time. Fourth, the cost-effectiveness and accessibility of PDT for atherosclerosis treatment pose additional considerations. The need for specialized equipment, trained personnel, and the cost associated with photosensitizer development could impact the widespread adoption of PDT. Comparative cost analyses and long-term efficacy studies will be essential to establishing PDT as a cost-effective alternative to current atherosclerosis treatments.

Despite these limitations, ongoing preclinical and clinical research efforts continue to explore PDT’s therapeutic potential in atherosclerosis. Innovations in photosensitizer chemistry, light delivery technologies, and treatment protocols are pivotal for overcoming current challenges. Furthermore, integrating PDT with existing atherosclerosis management strategies, such as statin therapy or mechanical interventions, may offer synergistic effects, enhancing treatment outcomes and patient benefits.

In conclusion, while PDT presents a promising avenue for atherosclerosis treatment, addressing its limitations through dedicated research and technological advancements is imperative. The selective ablation of atherosclerotic plaques, coupled with the potential for inducing immunomodulatory and plaque-stabilizing effects, underscores PDT’s therapeutic promise. Continued research, optimization of treatment parameters, and comprehensive clinical trials will be crucial for realizing the full potential of PDT in combating atherosclerosis, aiming to reduce the global burden of cardiovascular diseases.

## Figures and Tables

**Table 1:** Detection and clinical application of atherosclerotic plaque characteristics using imaging technologies.

Imaging Technologies		Fibrous Cap	Lipid Core	Macrophages	Calcification	Thrombus	Lumen Integrity	Angiogenesis	Plaque Burden
Non-invasive Technologies	Doppler ultrasound (carotid/femoral arteries)	−	−	−	−	−	−	+	+
	Coronary CT Angiography (CCTA)	−	+	−	+	−	+	−	+
	Coronary artery calcium score (CAC)	−	−	−	+	−	−	−	−
Invasive Technologies	Coronary Angiography (CAG)	−	−	−	−	−	+	−	−
	Intravascular Ultrasound (IVUS)	+	+	−	+	+	+	−	+
	Virtual Histology-Intravascular Ultrasound (VH-IVUS)	−	+	−	+	−	+	−	+
	Angioscope	+	+	−	−	+	+	−	−
	Optical coherence tomography (OCT)	+	+	+	+	+	+	+	−
	Near infrared spectroscopy (NIRS)	+	+	+	+	−	−	−	−
Emerging Technologies	Magnetic resonance angiography (MRA)	−	−	−	−	+	+	−	−
	Positron emission computed tomography (PET)	−	−	+	+	−	−	−	−
